# Impacts of the COVID-19 Pandemic on the Bereaved: A Study of Bereaved Weibo Users

**DOI:** 10.3390/healthcare9060724

**Published:** 2021-06-12

**Authors:** Nuo Han, Gewei Chen, Sijia Li, Feng Huang, Xiaoyang Wang, Xiaopeng Ren, Tingshao Zhu

**Affiliations:** 1 CAS Key Laboratory of Behavioral Science, Institute of Psychology, Chinese Academy of Sciences, Beijing 100101, China; hann@psych.ac.cn (N.H.); lisj@psych.ac.cn (S.L.); huangf@psych.ac.cn (F.H.); wangxiaoyang@psych.ac.cn (X.W.); 2 Department of Psychology, University of Chinese Academy of Sciences, Beijing 100049, China; 3 Beijing Key Laboratory of Applied Experimental Psychology, National Demonstration Center for Experimental Psychology Education, Faculty of Psychology, Beijing Normal University, Beijing 100875, China; 201711061108@mail.bnu.edu.cn

**Keywords:** public health, social media, OER, bereavement, mental health, COVID-19

## Abstract

The global COVID-19 pandemic may significantly affect the experiences of death and bereavement. This study aimed to learn from recent outbreaks of infectious diseases and further understand their impacts on bereavement. We obtained psychological status scores for 32 individuals bereaved due to COVID-19 and 127 individuals bereaved due to non-COVID-19 causes using the online ecological recognition (OER) approach. Next, a sentiment analysis and independent sample t-test were performed to examine the differences between these two groups. The results indicated that the individuals bereaved due to COVID-19 were more insecure and more preoccupied with the grief of the moment than those bereaved due to non-COVID-19 reasons, while the latter group had higher depression scores than the former group. This study can guide policy-makers and clinical practitioners to provide more targeted and sustainable post-bereavement support for both bereaved groups during the COVID-19 period.

## 1. Introduction

The coronavirus disease 2019 (COVID-19) pandemic has become a global public health crisis and is a leading cause of death worldwide [1,2]. As of 15 March 2021, China’s death toll had surpassed 4600, while the global death toll had surpassed 266,000; these numbers continue to increase. Researchers have found that for each COVID-19 death, approximately nine close family members suffered the loss of a loved one [3]; thus, there are many bereaved and numerous individuals are undergoing bereavement in this pandemic in China and across the world.

Bereavement, which is defined as the situation of having recently lost a significant person through death [4], has been shown to increase the risk of mental health problems, such as depressive symptoms, major depressive episodes [4,5,6,7,8,9], and anxiety-related disorders [4,8,9,10,11,12,13]. Bereavement has also been associated with other psychological symptoms, such as anger [7,14,15,16], fear [9,17], grief [7,16,18,19], and subjective well-being [20,21]. Researchers have found that prolonged grief, post-traumatic stress, anxiety, and depressive symptom levels are elevated among Chinese people that have been bereaved due to COVID-19 [22].

Losing a loved one due to COVID-19 makes this type of bereavement different in critical aspects from other types of bereavement [23,24,25]. First, families bereaved due to COVID-19 were unable to care for their family members and be with dying loved ones [26]. Second, these families could neither say goodbye to their loved ones nor express and process their grief through funeral ceremonies [23,27,28]. In addition, these families had to experience the fear of being infected [26,29] and exposure to stigma and social discrimination [23,26,29]. These reasons led to the unique experiences of COVID-19 bereavement compared with normal bereavement.

Studies have found individuals bereaved because of death due to a pandemic may experience subsequent mental health problems [24,25,30,31,32,33]. Eisma et al. found higher grief levels in individuals bereaved due to COVID-19 than in those who experienced natural loss [34]. This finding might indicate that COVID-19-related deaths could potentially increase the risk of an adverse outcome in terms of bereavement [35]. Additionally, learning from the impacts of COVID-19 on the bereaved would be pertinent for bereavement support during the pandemic [35]. Understanding the impacts is important because this information might be applied to support bereaved families and to inform service developments for the provision of ongoing post-bereavement support [9,24,35,36]. Research in this area is limited [35]; thus, in this study, we explored the differences in mental health between those that have been bereaved due to COVID-19 and to causes other than COVID-19, so as to provide more efficiently support those bereaved due to COVID-19.

A traditional research method to measure psychological impacts is through a self-report questionnaire; however, it was difficult to conduct traditional surveys during the ongoing COVID-19 epidemic. Online surveys rely on the cooperation of the participants and may cause extra burdens. During the COVID-19 pandemic, individuals have widely used online social networks (OSNs) to express their thoughts and feelings [37,38,39,40]. The ubiquity of OSNs provided an opportunity for this study. We selected Sina Weibo, a leading Chinese OSN with more than 516 million registered users, as an analytics platform [41]. All microblogs on Sina Weibo are publicly available and can be used to recognise individual psychological status and ascertain mental health statuses [39,42,43], analyse emotional states [39,44], and apply cognitional tests [39,43,44]. OSN data make real-time, non-invasive detection possible, ensuring the objectivity, timeliness, and continuity of the data. 

In this study, we accessed the OSN data for two bereaved groups in China: one group bereaved due to COVID-19 and one group bereaved due to non-COVID-19 reasons. The purpose of this study was to find the differences in psychological impacts between the two bereaved groups, so as to improve the targeted mental health care for individuals bereaved due to COVID-19. This study could also provide support for ongoing post-bereavement due to COVID-19.

## 2. Materials and Methods

### 2.1. Participants and Data Collection

Online samples were used to investigate the differences in psychological impacts between two groups of bereaved: one group bereaved due to COVID-19 and one group bereaved due to non-COVID-19 reasons. The sample included 159 bereaved individuals (30 male, 129 female). We used the date when the National Health Commission of China officially identified COVID-19 as a B-type infectious disease, 20 January 2020, as the starting point for COVID-19 in China [45]. Considering the number of newly confirmed cases is no longer increasing explosively in China, since March 2020, the pandemic situation has been considered to have stabilised. We collected original posts published on Weibo from 20 January 2020 to 1 March 2020. Such original posts are spontaneous, with the intent to communicate or share a given experience in virtual social media. Privacy protection was strictly ensured during the study, in line with the ethical principles listed by Kosinski, Matz, Gosling, Popov, and Stillwell [46]. The research protocol was approved in advance by the Ethics Committee of the Institute of Psychology, Chinese Academy of Sciences (approval number: H15009).

The Weibo posts by the bereaved were selected according to the following steps.

First, we searched for six-word combinations, namely, ‘pandemic + farewell’, ‘pandemic + passed away’, ‘pandemic + dead’, ‘COVID-19 + farewell’, ‘COVID-19 + passed away’, and ‘COVID-19 + dead’ in Weibo users’ microblog content. We found 282 Weibo users whose original posts contained such bereavement information.

Second, to ensure that the cause of death was clearly mentioned in a user’s Weibo messages, researchers manually checked all collected posts. We also ensured that the deceased had a clear affective bond with the Weibo user and distinguished whether the user was bereaved due to COVID-19. In this step, we selected 159 Weibo users who mentioned the illness of a family member and their subsequent death. There were 32 Weibo users in the COVID-19 bereavement group (CB group) and 127 Weibo users in the non-COVID-19 bereavement group (NCB group). The gender and age distributions for the users were similar in both groups. The remaining 123 Weibo users did not fulfil the conditions for losing relatives or specifying the cause of death.

After identifying the 159 bereaved users and the dates of the deaths from which they were grieving, to conduct the analysis, we retrieved these users’ profile information and their Weibo messages for the four weeks after the bereavement date.

### 2.2. Measures and Analysis

#### 2.2.1. Data Information

A web crawler was used to download all of the users’ profile information, microblogging behaviour, and Weibo messages from Sina Weibo. The user profile information included a user’s age, gender, and self-defined location. The micro-blogging behaviours include a user’s tag, post count, friend count, and follower count. Weibo messages include the user ID, the time of the post, and the text. 

#### 2.2.2. Measurement of Psychological Status

Instead of self-reporting, we used online ecological recognition (OER) [43], a method used for the automatic recognition of psychological status (e.g., well-being and grief) [42,47,48,49,50]. Automatic recognition is based on validated prediction models that predict a user’s mental status on the basis of the dynamic features of an OSN. Dynamic features refer to those features showing obvious changes over time (e.g., per day), which are relative to static features. In this study, the meaning of ‘dynamic features’ is a user’s daily microblog updates on Weibo [48]. When a user posted on Weibo, their online data changed and their dynamic feature value was correspondingly altered.

For each user, we extracted dynamic features from two categories: text features and behavioural features [51,52]. Text features were based on the linguistic inquiry and word count (LIWC) approach, which is widely used in natural language processing for mapping psychological and linguistic dimensions of written expression [53]. In this research, we used the Simplified Chinese microblog word count (SCWBWC) approach for analysis. The SCWBWC approach was established according to the LIWC dictionary and the traditional Chinese version of an LIWC (CLIWC) dictionary. Next, high-frequency words on Weibo were added into the Simplified Chinese version of LIWC. This approach is promising for psychological and other types of research based on Weibo [52]. The SCWBWC reports the degree of Simplified Chinese usage in 91 dimensions (e.g., ‘negative emotion words’ and ‘death words’). Then, we extracted 11 behavioural features: ‘counts of words’, ‘counts of words per sentence’, ‘counts of URLs’, ‘counts of @ names’, ‘counts of tags’, ‘counts of posts’, ‘counts of original posts’, ‘counts of comments’, ‘counts of positive emotion emoji use’, ‘counts of negative emotion emoji use’, and ‘counts of neutral emotion emoji use’.

**Figure 1:**
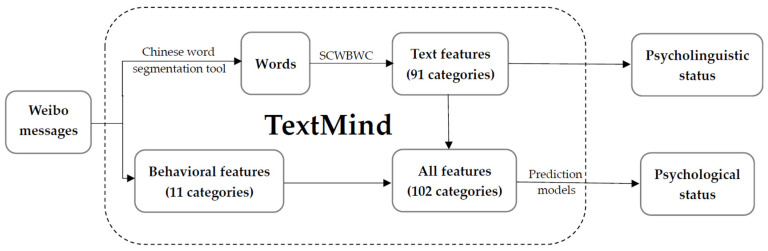
Measurement of psycholinguistic status and psychological status.

Because some users did not post new posts daily, we aggregated the text and behavioural features of the CB and NCB groups. Next, we used the Chinese version of the text analysis software Textmind to calculate all 102 features [52]. Textmind divides a microblog into several word pieces according to SCWBWC (e.g., ‘I lost my loved one’ to ‘I’, ‘lost’, ‘my’, ‘loved’, ‘one’) and then calculates the frequency of word pieces of each SCWBWC category. During the last step, Textmind output the ratio for the four weeks as the input dynamic features of each user and fed them into the prediction models. Figure 1 depicts the procedure from data extraction to psychological status quantification.

The prediction models use machine learning algorithms to map dynamic features to related questionnaire scores, for example emotional indices (e.g., anxiety and depression) and cognitive indices (e.g., social risk judgement and life satisfaction). Figure 1 also depicts the procedure from feature extraction to psychological status. Previous studies calculated the Pearson correlation coefficients between the predicted and questionnaire scores [42,47,49,52,54]. The correlation coefficients across all dimensions were in the range of 0.45–0.58, indicating a moderate level.

#### 2.2.3. Procedure

In this study, we recorded the timestamps for first reporting bereavement as a boundary and then measured the psychological status of each user. Because the bereavement timestamps differed by user, for each user we assessed their psychological status 4 weeks after the incident. The measurement employed the data for the 4 weeks after bereavement. To explore the differences between the CB group and the NCB group, we performed independent sample t tests on bereavement users by using Statistical Product and Service Solutions (SPSS) 22 [55]. The dependent variables were the OER-predicted scores for emotions and cognition and the SCWBWC word frequency.

## 3. Results

### 3.1. Demographics

Among the 159 bereavement users, 20% people suffered from COVID-19 bereavement and 81% were female. Sixteen percent of them registered their location as Hubei Province in their profile, which was not only the first province to find COVID-19 cases, but also the most severely affected province in China. The demographic profiles of these groups are shown in Table 1.

### 3.2. Linguistic Difference

In this study, we compared the word frequency rates of SCWBWC categories between CB and NCB people, as shown in Table 2. We analysed two types of SCWBWC categories: words of concerns and words of time. Categories in words of concerns included achievement (e.g., work, lose, and successful), death (e.g., coffin, bury, and kill), health (e.g., clinic, flu, and pill), leisure (e.g., cook, chat, and movie), family (e.g., daughter, husband, and wife), friends (e.g., buddy, friend, and neighbour), religion (e.g., grace, church, and bless), money (e.g., poor, generous, and rich), and love (e.g., rose, groom, and kiss). The words of concerns reflect what people are paying attention to. Categories in words of time included the past state (e.g., yesterday, already, and past), present state (e.g., today, present, and now), and future state (e.g., tomorrow, afterlife, and will). The words of time reflect the time state of people’s attention.

The results showed that CB people used significantly more death words (*t*(157) = 2.17, *p* = 0.036, *d* = 0.47), significantly more achievement words (*t*(157) = 1.79, *p* = 0.075, *d* = 0.33), but significantly fewer love words (*t*(157) = −1.11, *p* = 0.065, *d* = −0.27) than NCB. CB people also used significantly more present state words (*t*(157) = 1.95, *p* = 0.053, *d* = 0.32) NCB. Other categories showed no significant differences between CB and NCB people.

### 3.3. Emotional Indices

In order to explore the emotions of bereavement for individuals during the epidemic and whether the negative emotions of the people who had been bereaved by COVID-19 were worse than for the non-COVID-19 group, we compared the emotions between two types of bereavement. As shown in Table 3, the results indicated that CB people had significantly lower scores for negative emotional indices of psychological status in terms of stress (*t*(157) = 0.79, *p* = 0.162, *d* = −0.30) than NCB. Other negative emotional indices of psychological status showed no significant differences between the CB group and NCB group.

### 3.4. Cognitive Indices

We also compared cognition between the two bereavement groups. Collective behaviour intention represents the intention to adopt collective behaviour in order to change the group status. Positive relationship represents the ability to build good and effective relationships with others. Life goals represent the ability of individuals to set goals for their own lives and to stick to these goals. Life satisfaction represents an individual’s comprehensive cognitive evaluation of whether they are satisfied with their life state based on the standards set by themselves.

As shown in Table 4, the results indicate that CB people had significantly higher scores for cognitive indices of psychological status for collective behaviour (*t*(157) = 1.92, *p* = 0.057, *d* = 0.37) but had significantly lower scores of cognitive indices of psychological status for life goals (*t*(157) = −1.86, *p* = 0.067, *d* = −0.31) than NCB. Other cognitive indices of psychological status showed no significant difference between CB and NCB groups.

## 4. Discussion

The devastating impacts of COVID-19 worldwide have been well documented, with a high number of deaths recorded. This study explored the impacts of death due to COVID-19 on the bereaved via Sina Weibo. We compared the CB group and the NCB group, with the results indicating that although both groups were bereaved, some differences were observed in their psychological indices.

The findings demonstrated that the CB group used more achievement, death, love, and present state words than the NCB group did. The words of concern (achievement, death, and love) reflect attentional allocation, while the words of time (present state) reflect the temporal focus of attention. First, the greater use of achievement words means that the CB group became more focused on individual achievement than the NCB group. This finding might reflect a greater sense of security being needed in the wake of COVID-19 bereavements, as supported by previous studies [56,57]. According to the phaeton effect, the loss of a loved one can disrupt the rhythm of life, prompting the bereaved to pursue a sense of security and value, becoming a source of spiritual power for the pursuit of achievement, status, and wealth [58,59]. Second, the CB group used more death and present state words than the NCB group did. A previous study found that people who are going through bereavement are more focused on their present experience [60]. Similar to the previous study, this finding might also indicate that the CB group was immersed in the present moment of the death of their loved ones. This phenomenon might be due to the sudden, unexpected death of a loved one, with the bereavement more difficult to accept when individuals lack psychological anticipation [61,62]. Additionally, society, including the CB group members themselves, remains concerned about the pandemic, which consistently reminds them of the fact that their loved ones died of the pandemic. These reasons may make recovering from the setback of bereavement more difficult for the CB group than for the NCB group. This situation is also reflected in that the CB group used fewer love words than the NCB group did. The reason for this finding could be that the grief due to bereavement causes the CB group to devote less attention to maintaining romantic relationships than the NCB group.

In addition, Young et al. encouraged helping people bereaved by suicide to set goals for a new life [63]. As with acute bereavement, bereavement due to COVID-19 is also difficult to get over, which contributed to the CB group having lower life goal scores than the NCB group. Because the CB group had more difficulty recovering from the sudden death of a loved one, they had lower scores in terms of their ability to set goals for the future and achieve those goals than the NCB group did. Moreover, the infectivity of COVID-19 and the stigma from society might place the CB group in a vulnerable position. AS with the victims of an earthquake who developed a sense of relative deprivation, CB individuals have also developed group relative deprivation [64]. The CB group had higher scores for collective behaviour intention than the NCB group did [65].

The results also demonstrated that the NCB group had a higher score for depression than the CB group did. This result was beyond our expectation but was consistent with a previous study showing that people who were away from the COVID-19 risk centre showed stronger negative emotions than those in the risk centre [66]. There may be two main reasons for this result. First, deaths not due to COVID-19 were sometimes attributed to postponement of treatment of other life-threatening diseases or to avoiding health care facilities to prevent infection. This may have caused avoidable losses [62], contributing to the NCB group’s depression levels. Second, because of the grief reactions of the general public to the news of the continuing deaths, the NCB group might have been deprived of the psychological need to grieve alone during the acute grieving period. This may also have increased depression levels [67].

Based on our findings, clinical practitioners should focus on the loss of safety of the CB group and improve its sense of social identity to reduce the feelings of the group members relative deprivation [68]. To make the CB group feel cared for and relieve their insecurity, timely psychological support is very necessary. As resources could be particularly scarce during a serious pandemic situation, timely psychological support could also take many forms, including telemedicine and informal support groups [69]. Moreover, the negative effects of stigma on the CB group and the negative effects of sudden loss may also require attention. Clinical practitioners should encourage the CB group to look to the future and emphasise that life still goes on. Additionally, the depression levels of the NCB group should be considered. Policy makers should promote the provision and guarantee of medical facilities and public resources for non-pneumonia patients, even in special times, in order to reduce the psychological pressure on the NCB group. In addition, the CB group might require additional psychological support and encouragement to increase their sense of group identity [68].

Notably, some limitations should be considered when generalising this study’s findings. First, the veracity of the Weibo reports cannot be ascertained. This is an inevitable limitation for social media studies. Second, the sample size of this study was not large enough. In further studies, as the impact of the COVID-19 pandemic continues, we will try to expand the sample size and explore the psychological status of the CB group in other countries on other social media platforms (e.g., Twitter, Facebook). Third, the samples in this study may not be representative. There could be an element of selection bias, given that people who grieve in ‘public’ may have higher expressed emotions. We will use a combination of online and offline sampling methods in future studies, which could make the sampling more comprehensive.

## 5. Conclusions

Previous studies in the literature have focused on survivors of illness and have not investigated outcomes and support for the bereaved during a pandemic. According to our literature review, no empirical study had explored the psychological status of bereavement due to COVID-19. In this study, we assessed the psychological impacts on the CB group by comparing it with that of the NCB group. The results indicated that the CB group was more insecure and more preoccupied with the grief of the moment than the NCB group was, while the NCB group had higher depression scores than the CB group; thus, society should provide targeted support to both groups. Additionally, social media data can be used to obtain a timely understanding of the impacts of public health emergencies on the mental health of specific groups, including during a pandemic.

## Figures and Tables

**Table 1:** Demographic profiles of bereavement users.

Class	Type	CB Group *n* (%)	NCB Group *n* (%)
Gender	Male	4 (12.50)	26 (20.47)
	Female	28 (77.50)	101 (79.53)
Age	20~29	19 (59.38)	60 (47.24)
	30~39	9 (28.13)	41 (32.28)
	40~49	2 (6.25)	7 (5.51)
	50~	1 (3.13)	3 (2.36)
	Missing data	1 (3.13)	16 (12.60)
Location	Hubei Province	18 (56.25)	7 (5.51)
	Not Hubei Province	14 (43.75)	120 (94.49)
Total		32 (100)	127 (100)

Note: CB (COVID-19 bereavement) group is the group of people whose relatives died of COVID-19, while NCB (non-COVID-19 bereavement) group is the group of people whose relatives did not die of COVID-19.

**Table 2:** Linguistic Difference between CB group and NCB group.

Linguistic Use	CB Group (*N* = 32)		NCB Group (*N* = 127)		*t*	*p*	*df*
	*M*	*SD*	*M*	*SD*			
**Words of concerns**							
Achievement	10.59	9.53	7.82	7.35	1.79	0.075	157
Death	2.99	3.96	1.39	2.67	2.17	0.036 *	157
Health	8.53	10.32	6.43	8.82	1.16	0.247	157
Leisure	12.24	10.99	13.47	13.43	−0.48	0.630	157
Family	5.87	7.61	6.27	8.77	−0.23	0.816	157
Friends	0.63	1.00	0.91	1.45	−1.03	0.306	157
Religion	3.05	3.50	3.18	5.50	−0.13	0.895	157
Money	3.99	5.17	4.29	6.03	−0.25	0.801	157
Love	0.32	0.78	0.79	2.35	−1.11	0.065	157
**Words of time**							
Past state	2.08	3.20	1.67	2.75	0.72	0.473	157
Future state	1.92	6.77	1.26	3.75	0.74	0.459	157
present state	4.63	7.02	2.83	3.85	1.95	0.053	157

Note: CB (COVID-19 bereavement) group is the group of people whose relatives died of COVID-19, while NCB (non-COVID-19 bereavement) group is the group of people whose relatives died but did not die of COVID-19; *M* = mean; *SD* = standard deviation; *df* = degrees of freedom; * *p* < 0.05.

**Table 3:** Comparison of emotions between the CB group and NCB group.

Emotions	CB Group (*N* = 32)		NCB Group (*N* = 127)		*t*	*p*	*df*
	*M*	*SD*	*M*	*SD*			
Anxiety	4.20	1.20	4.58	1.49	−1.32	0.188	157
Depression	5.35	2.34	6.37	3.05	−1.77	0.079	157
Stress	4.04	2.51	5.00	3.69	−1.40	0.165	157
Grief	1.21	1.55	1.78	4.85	−0.66	0.511	157
Fear	0.52	1.04	0.32	0.68	1.02	0.313	157
Anger	1.94	0.68	1.78	0.45	−1.40	0.108	157

Note: CB (COVID-19 bereavement) group is the group of people whose relatives died of COVID-19, while NCB (non-COVID-19 bereavement) group is the group of people whose relatives died but did not die of COVID-19; *M* = mean; *SD* = standard deviation; *df* = degrees of freedom.

**Table 4:** Comparison of cognition between CB and NCB groups.

Cognition	CB Group (*N* = 32)		NCB Group (*N* = 127)		*t*	*p*	*df*
	*M*	*SD*	*M*	*SD*			
Collective behaviour intention	2.70	0.48	2.53	0.43	1.92	0.057	157
Positive relationship	12.04	1.67	12.22	1.68	−0.54	0.594	157
Life goal	12.02	1.05	12.47	1.74	−1.86	0.067	157
Life satisfaction	13.87	3.65	13.92	2.96	−0.09	0.929	157

Note. CB (COVID-19 bereavement) group is the group of people whose relatives died of COVID-19, and NCB (non-COVID-19 bereavement) group is the group of people whose relatives died but did not died of COVID-19; *M* = mean; *SD* = standard deviation; *df* = degrees of freedom.

## Data Availability

To protect the privacy of the participants, the original posts used for the analysis are not publicly available but are available from the corresponding author on reasonable request.
